# Can narrow-bandwidth light from UV-A to green alter secondary plant metabolism and increase *Brassica* plant defenses against aphids?

**DOI:** 10.1371/journal.pone.0188522

**Published:** 2017-11-30

**Authors:** Ole Rechner, Susanne Neugart, Monika Schreiner, Sasa Wu, Hans-Michael Poehling

**Affiliations:** 1 Section of Phytomedicine, Institute of Horticultural Production Systems, Hannover, Germany; 2 Department Plant Quality, Leibniz Institute of Vegetable and Ornamental Crops, Grossbeeren, Germany; Georg-August-Universitat Gottingen, GERMANY

## Abstract

Light of different wavelengths is essential for plant growth and development. Short-wavelength radiation such as UV can shift the composition of flavonoids, glucosinolates, and other plant metabolites responsible for enhanced defense against certain herbivorous insects. The intensity of light-induced, metabolite-based resistance is plant- and insect species-specific and depends on herbivore feeding guild and specialization. The increasing use of light-emitting diodes (LEDs) in horticultural plant production systems in protected environments enables the creation of tailor-made light scenarios for improved plant cultivation and induced defense against herbivorous insects. In this study, broccoli (*Brassica oleracea* var. *italica*) plants were grown in a climate chamber under broad spectra photosynthetic active radiation (PAR) and were additionally treated with the following narrow-bandwidth light generated with LEDs: UV-A (365 nm), violet (420 nm), blue (470 nm), or green (515 nm). We determined the influence of narrow-bandwidth light on broccoli plant growth, secondary plant metabolism (flavonol glycosides and glucosinolates), and plant-mediated light effects on the performance and behavior of the specialized cabbage aphid *Brevicoryne brassicae*. Green light increased plant height more than UV-A, violet, or blue LED treatments. Among flavonol glycosides, specific quercetin and kaempferol glycosides were increased under violet light. The concentration of 3-indolylmethyl glucosinolate in plants was increased by UV-A treatment. *B*. *brassicae* performance was not influenced by the different light qualities, but in host-choice tests, *B*. *brassicae* preferred previously blue-illuminated plants (but not UV-A-, violet-, or green-illuminated plants) over control plants.

## Introduction

Arthropod pests like aphids (Hemiptera: Aphididae) can damage horticultural plants by removing assimilates (phloem-feeding), producing honeydew, and transmitting viruses [1]. The effect of aphids and other arthropod pests on plants can be affected by light quality [2]. UV-B radiation, for example, increases the biosynthesis of protective phenolic compounds like kaempferol and quercetin glycosides in plants [3–4]. Furthermore, the concentration of specific glucosinolates in *Brassica oleracea* var. *italica* P. (Brassicaceae; broccoli) can be increased by treatment with UV-B or UV-A radiation [5–6]. This light-induced increase in plant metabolites results from the stimulation of specific photoreceptors followed by the activation of a signal transduction chain and the triggering of transcription factors and genes involved in secondary metabolite biosynthesis [2]. Expression of these genes shifts the composition of metabolites in the plant, and changes in specific secondary plant metabolites can enhance or decrease the susceptibility to certain herbivorous insects [2, 7]. In addition to UV-B and UV-A, other light qualities, e.g., blue, green, and red, may also induce the biosynthesis of certain plant metabolites such as flavonoids and glucosinolates and alter the resistance of the plant [8–13].

With the increasing development of LED technology and its use in horticultural production systems in protected environments, plant producers are now able to create specific light scenarios for influencing plant growth and quality and also plant metabolites so as to protect plants against herbivorous insects [14–15]. The application of LED-generated narrow-bandwidth light of different quality is a promising approach for enhancing the production of secondary metabolites in plants [16].

The effect of LED light treatments on plants is receiving increasing attention from researchers. Additional green light generated with LEDs did not influence the growth of *Cucumis sativus* L. (Cucurbitaceae; cucumber seedlings) [17]. In contrast, green LED light promoted growth of *Lactuca sativa* L. (Asteraceae; lettuce) [18]. Increases in blue light intensity enhanced the chlorophyll content per leaf area and photosynthetic rate in cucumber, resulting in improved primary plant metabolism and growth [17, 19].

LED lighting can also affect concentration of secondary plant metabolites. Artificial LED lighting enriched with blue light enhanced the growth and increased the total phenolic content of *Ocimum basilicum* L. (Lamiaceae; basil) compared to broad spectra fluorescent light [20]. Treatment of *Brassica rapa* ssp. *pekinensis* (Brassicaceae; Chinese cabbage) with blue LED light also increased the biosynthesis of phenylpropanoids including quercetin and kaempferol glycosides [11]. Furthermore, *Cardamine fauriei* Maxim. (Brassicaceae; Ezo-wasabi in Japanese) contained increased concentrations of aliphatic glucosinolates after irradiation with blue and red LED light [12]. Dader et al. [8] irradiated two plant species with artificial UV-A and induced flavonoids in *Capsicum annuum* L. (Solanaceae; pepper) but not in *Solanum melongena* L. (Solanaceae; eggplant), indicating species-specific reactions to various light treatments. Green and yellow lights enhanced production of total phenolics and total flavonoids in callus cultures of *Prunella vulgaris* L. (Lamiaceae; self-heal) [21].

By altering plant metabolites, light can affect the behavior, performance, and development of herbivorous insects [2, 7, 22]. Treatment with UV-B increased the concentrations of kaempferol glycosides and specific glucosinolates in broccoli plants, and feeding on these plants reduced the fecundity of the specialist aphid *Brevicoryne brassicae* Linnaeus but improved the performance of the generalist aphid *Myzus persicae* Sulzer (both Hemiptera: Aphididae) [6]. Moreover, *B*. *brassicae* preferred to colonize broccoli plants grown under ambient UV conditions than under low-UV conditions in open, plastic tunnels equipped with UV-blocking vs. UV-transmitting films but *B*. *brassicae* population growth was reduced on plants grown under high-UV conditions [22–25]. This indicates that insect reactions to light treatments can be insect-specific.

To our knowledge, no study has compared the effects of short-wavelength light (such as UV-A) and longer wavelength light (violet to green in the visible spectrum) on plant growth, plant metabolic composition, and specialized herbivorous insects in protected horticultural production systems. In this study, we tested the hypothesis that different LED-generated light qualities (ranging from UV-A to green) can alter the growth as well as the metabolic composition (flavonoids and glucosinolates) of *Brassica oleracea* var. *italic* (broccoli) plants and indirectly influence (via plant metabolites) the choice of host plant and performance of the cabbage aphid *B*. *brassicae*.

## Material and methods

### Rearing of insects

Cabbage aphids (*B*. *brassicae*) were collected outdoors from broccoli plants at the Leibniz University Hannover, Institute of Horticultural Production Systems, Section of Phytomedicine, Hannover (N 52° 23`39.22”, E 9° 42`18.86”). The aphids were reared on 4-week-old broccoli plants in a gauze cage with a wooden frame (85 cm × 60 cm × 60 cm); the cages were kept in a climate chamber (20 ± 2°C, relative humidity 65 ± 10%, photoperiod 16:8 h L:D). Every week, half of the plants in each cage were replaced to continuously provide a high quality food source. Adult aphids used in experiments were randomly collected from these cages.

### Plant material and growth conditions

Broccoli plants [*B*. *oleracea* var. *italica*, cv Monopoly; F1 Hybrid; Syngenta Enkhuizen, Netherlands] were grown under specific light conditions (see Experimental layout and light treatments) from seeds in pots (12 cm diameter, 9 cm height, one seedling per pot) containing fertilized soil (Fruhstorfer Erde Type P, Hawita Gruppe, Vechta, Germany) for 4 weeks. Four-week-old broccoli plants were used for the experiments with aphids.

### Experimental layout and light treatments

**Fig 1:**
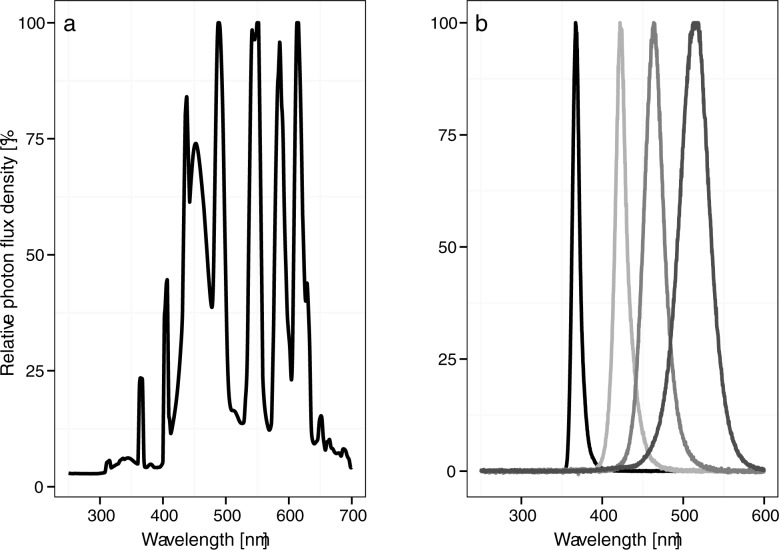
Wavelength [nm] spectra and corresponding photon flux density [%] for (a) Osram 840 fluorescent tubes (Lumilux Interna L 58 W / T8, 840, 5200 lm, 4000 K) and for (b) hexagonal 1-W high-power single-chip LED emitters (H2A1-H365-E, H2A1-H420, H2A1-H470, H2A1-H515) used in the climate chamber.

The experiments were conducted in a climate chamber (Viessmann, 4 m x 3 m x 2.40 m, Allendorf, Germany) with the following conditions: temperature 20 ± 2°C, relative humidity 70 ± 10%, and photoperiod 16:8 h L:D. The climate chamber contained five metal tables that were covered with black mulch film (PP-Gewebe, supplied by Raiffeisen GmbH, Bad Zwischenahn, Germany). On the tables in the chamber, 20 compartments (0.75 m x 0.3 m x 1 m) were separated by wooden frames covered with reflective mulch film (full metal on black film, supplied by Sunup Reflective Films/Star Metal Plating, Escondido, California USA) to prevent light interference from neighboring areas; the tops of the compartments were not closed or covered. The chamber was illuminated with 50 fluorescent tubes (Osram Lumilux Interna, L 58 W / T8, 840, 5200 lm, 4000 K, Munich, Germany), which were mounted 1 m above the tables to provide equal photon flux densities of photosynthetic active radiation (PAR). Additional light treatments with specific narrow-bandwidth wavelengths were generated with hexagonal 1-W high-power single-chip LED emitters. For each LED illuminated compartment, two small aluminum plates (25 cm x 5 cm) were each equipped with three high-power LEDs. The aluminum plates were separated by 5 cm to ensure minimal shading for PAR radiation. The intensities of the high-power LEDs were regulated with rotary potentiometers by high-power LED drivers (LED-Slave, PWM Dimmer Onboard, PCB Components, Hildesheim, Germany). The LED panels were located 12 cm above the plants. The peak wavelengths of the LEDs were UV-A 365 nm (H2A1-H365-E), violet 420 nm (H2A1-H420), blue 470 nm (H2A1-H470), and green 515 nm (H2A1-H515). The LEDs were supplied by Roithner Laser Technik GmbH, Vienna, Austria. The radiation spectra of the LEDs and the light tubes were measured with a UV/VIS fiber and a compatible fiber optic spectrometer (AvaSpec 2048–2, supplied by AVANTES, Appeldoorn, The Netherlands) (Fig 1). PAR was measured with a Licor LI-250-A light meter (Lincoln, Nebraska USA) and was adjusted to 100 ± 10 μmol m^-2^ s^-1^ in all treatments such that 50% was generated by violet, blue, or green LEDs (Table 1). The UV-A intensities were measured in W/m^2^ and μW/cm^2^, respectively, with an ALMEMO 2390–5 spectra radiometer (Ahlborn Mess- und Regelungstechnik GmbH, Holzkirchen, Germany) and light intensities were comparable among all treatments. Intensities were converted to photon flux density (μmol m^-2^ s^-1^) based on the spectrum, Planck’s constant, and Avogadro’s number. For the UV-A treatment, the energy of the light source (kJ m^-2^ s^-1^) was also determined so that the results could be compared with those of other studies (Table 1). Each light compartment contained six broccoli plants, and each light treatment was represented by four replicate light compartments. Thus, the climate chamber contained 120 plants.

**Table 1:** Light intensities provided by the four light treatments with LEDs and the control. The background and control illumination was provided by Osram 840 fluorescent tubes.

	Measured light intensities			
Light treatment	PAR Osram 840 (400–700 nm) [μmol m^-2^ s^-1^]	PAR LEDs (400–700 nm) [μmol m^-2^ s^-1^]	UV-A (315–380 nm) [μmol m^-2^ s^-1^]	UV-A (315–380 nm) [kJ m^-2^ d^-1^]
Control	100 ± 10	0	0	0
UV-A 365 nm	100 ± 10	0	61 ± 3	11520
Violet 420 nm	50 ± 5	50 ± 5	0	0
Blue 470 nm	50 ± 5	50 ± 5	0	0
Green 515 nm	50 ± 5	50 ± 5	0	0

### *B*. *brassicae* performance experiment

For determination of aphid performance, a brush was used to carefully place 12 *B*. *brassicae* adults on the underside of the second leaf of two of the six plants per light compartment. The leaf with aphids was enclosed in a 3-cm-diameter clip cage attached to the underside of the leaf and not directly exposed to the different light treatments to exclude direct light effects on the aphids. The adult aphids were allowed to deposit larvae for 24 h before they were removed. Twelve larvae were permitted to develop per plant (per clip cage). The number of days required for the larvae to develop into adults was recorded (developmental time), and the final weights of 10 adults per clip cage were measured with a microbalance (Type MC 5 Sartorius, Goettingen, Germany). Two adults per plant were kept separately in clip cages on the same plants (second and third leaf), and their offspring were counted every second day to measure fecundity [Md].

### *B*. *brassicae* host selection experiment

Choice experiments were designed to investigate the behavioral response of *B*. *brassicae* to plants grown with the five light treatments. Plants were grown for 4 weeks under fluorescent tubes (Osram Lumilux Interna L 58 W / T8, 840, 5200 lm, 4000 K, control conditions) in the climate chamber and were additionally treated with UV-A 365 nm, violet 420 nm, blue 470 nm, or green 515 nm or received no additional light treatment. These plants were used for the host selection experiment, which was carried out under usual broad spectra illumination in the climate chamber and not under the specific light treatments to avoid direct and visual effects of the light treatments on the aphids’ behavior. The experiment used three arenas. One release arena for *B*. *brassicae* was located in the middle, one arena for a plant leaf was located on the left side of the release arena, and one arena for another plant leaf was located on the right side of the release arena. All three arenas were connected by holes so that *B*. *brassicae* was able to walk between the leaves. Twenty synchronized *B*. *brassicae* adults were released in the middle arena and always had the choice between the leaf of one light-induced and one control plant; these leaves were randomly located in the left or the right arena and still attached to the living plant during the experiment. The choice experiment was run for 20 h. Each comparison of control leaf vs. light-induced leaf was represented by 10 replicate assays in each of two arena systems, resulting in the testing of a total of 200 *B*. *brassicae* per treatment.

### Effects of light treatments on plant leaf number, height, and weight without aphid infestation

To determine how the five light treatments affected plant morphology, additional plants were grown without aphids for 4 weeks under the same conditions described above. A total of 16 4-week-old broccoli plants for each light treatment (four from each replicate compartment) were randomly selected and destructively sampled for biomass analysis. Leaves were counted, and plant height was measured from the main stem base to the top of the plants. After the harvested plants were kept at 65°C for 5 days, their dry weights were determined with an electronic balance (Type BP 3100 P, Sartorius, Goettingen, Germany).

### Sample preparation for metabolite analysis

For each light treatment, leaves were collected from eight 6-week-old broccoli plants (two per replicate compartment) that were infested or not infested with *B*. *brassicae*. Each infested plant had two infested leaves in clip cages as described earlier. The non-infested leaves were obtained from separate plants, i.e., plants without infestation. A mixed sample of all leaves per plant (excluding stems and midribs) was placed in liquid nitrogen, freeze-dried for 5 days (using a Christ Alpha 1–4 LSC freeze drier), and subsequently ground to a powder (≤ 0.25 mm).

#### Flavonoid analysis

Flavonoids were analyzed according to Schmidt et al. [26] with modification. Lyophilized broccoli tissue (0.02 g) was extracted with 600 μl of 60% aqueous methanol on a magnetic stirrer plate for 40 min at 20°C. The extract was centrifuged at 4500 rpm for 10 min at the same temperature, and the supernatant was collected in a reaction tube. This process was repeated twice with 300 μl of 60% aqueous methanol for 20 min and 10 min, respectively; the three supernatants per sample were combined. The extract was subsequently evaporated until it was dry and was then suspended in 200 μl of 10% aqueous methanol. The extract was centrifuged at 3000 rpm for 5 min at 20°C through a Corning® Costar® Spin-X® plastic centrifuge tube filter (Sigma Aldrich Chemical Co., St. Louis, MO, USA) for HPLC analysis. Each extraction was carried out in duplicate.

Flavonoid composition (including hydroxycinnamic acid derivatives and glycosides of flavonols) and concentrations were determined using a series 1100 HPLC (Agilent Technologies, Waldbronn, Germany) equipped with a degaser, binary pump, autosampler, column oven, and photodiode array detector. An Ascentis^®^ Express F5 column (150 mm × 4.6 mm, 5 μm, Supelco) was used to separate the compounds at 25°C. Eluent A was 0.5% acetic acid, and eluent B was 100% acetonitrile. The gradient used for eluent B was 5–12% (0–3 min), 12–25% (3–46 min), 25–90% (46–49.5 min), 90% isocratic (49.5–52 min), 90–5% (52–52.7 min), and 5% isocratic (52.7–59 min). The determination was conducted at a flow rate of 0.85 ml min^-1^ and a wavelength of 320 nm, 330 nm, and 370 nm for hydroxycinnamic acid derivates, acylated flavonol glycosides, and non-acylated flavonol glycosides, respectively. The hydroxycinnamic acid derivatives and glycosides of flavonols were identified as deprotonated molecular ions and characteristic mass fragment ions according to Schmidt et al. [26] by HPLC-DAD-ESI-MS^n^ using an Agilent series 1100 ion trap mass spectrometer in negative ionization mode. Nitrogen was used as the dry gas (10 L min^-1^, 325°C) and the nebulizer gas (40 psi) with a capillary voltage of -3500 V. Helium was used as the collision gas in the ion trap. The mass optimization for the ion optics of the mass spectrometer for quercetin was performed at *m/z* 301 or arbitrarily at *m/z* 1000. The MS^n^ experiments were performed in auto up to HPLC-DAD-ESI-MS^3^ in a scan from *m/z* 200–2000. Standards (chlorogenic acid, quercertin 3-glucoside, and kaempferol 3-glucoside; Roth, Karlsruhe, Germany) were used for external calibration curves. Results are presented as μg g^-1^ dry weight. Flavonol glycoside and hydroxycinnamic acid derivative concentrations were determined for four replicate light compartments per treatment with two broccoli plants per replicate compartment; each replicate sample was measured in duplicate.

#### Glucosinolate analysis

Glucosinolate concentration was determined as desulfo-glucosinolates using a modified method according to Wiesner et al. [27]. A 20.0-mg quantity of powdered sample plus 100 μl of 0.1 mM 2-propenyl glucosinolate (BCR-367R, Community Bureau of Reference, Brussels, Belgium) as the internal standard was extracted with 750 μl of 70% (v/v) methanol at 70°C. The preparation was boiled for 10 min and then centrifuged (2250 g) for 5 min at room temperature. The supernatant was decanted, and the residue was re-extracted twice with 500 μl of hot 70% methanol each time. The pooled extracts were loaded onto a mini column containing 500 μl of DEAD-Sephadex A-25 that had been conditioned with 2 M acetic acid and washed with 6 M imidazole formate. After loading, the column was washed with 0.02 M sodium acetate buffer. Finally, 75 μl of an aryl sulfatase solution (Sigma-Aldrich, Steinheim, Germany) was added, and the preparation was incubated overnight. Desulfo-glucosinolates were eluted with water and analyzed by HPLC using a Merck HPLC system (Merck-Hitachi, Darmstadt, Germany) with a Spherisorb ODS2 column (Bischoff, Leonberg Germany; particle size 5 μm, 250 mm x 4 mm). HPLC conditions were as follows: solvent A, MilliQ water; solvent B, 20% v/v acetonitrile in MilliQ water; solvent C, 100% acetonitrile. The 60-min run consisted of 1% (v/v) B (2 min), 1% to 20% (v/v) B (34 min), a 6-min hold at 20% (v/v) B, 20% B to 100% (v/v) C (2 min), a 5-min hold at 100% (v/v) C, 100% (v/v) C to 1% (v/v) B (2 min), and finally a 10-min hold at 1% (v/v) B. Determination was conducted at a flow rate of 0.7 ml min^-1^ and a wavelength of 229 nm. Desulfo-glucosinolates were identified based on comparison of retention times and UV absorption spectra with those of known standards. Additionally, desulfo-glucosinolates were previously identified in other *Brassica* species by HPLC-ESI–MS^2^ using Agilent 1100 series (Agilent Technologies, Waldbronn, Germany) in positive ionization mode [28–29]. Glucosinolate concentration was calculated using 2-propenyl glucosinolate as an internal standard and the response factor of each compound relative to 2-propenyl glucosinolate [30]. Results are presented as μg g^-1^ dry weight. Glucosinolate concentration was determined in four replicate light compartments per treatment with two broccoli plants per replicate compartment; each replicate sample was measured in duplicate.

### Statistical analysis

The data were analyzed in R 2.15.2 [31]. Graphs were made with the package ggplot2 [32]. The effects of the light treatments on plant height, plant dry weight, and aphid adult weight were analyzed using generalized linear mixed models (GLMM) followed by Tukey *post hoc* tests [33].

The effects of aphid infestation and light treatments on plant secondary metabolites (concentrations of flavonoids and glucosinolates) were also analyzed using GLMM and the package lsmeans by estimating least-squares means and differences of contrast. Differences between single light treatments and the control were subsequently determined with a Tukey *post hoc* test. Effects of light treatments were averaged over the two levels of infestation (control without aphids and plants infested with *B*. *brassicae*), and effects of infestation were averaged over the levels of variant. The total numbers of offspring and the developmental time of aphids as well as the leaf number per plant were analyzed by generalized linear models (GLM) using a log-link together with a quasi-Poisson distribution. The effects of light treatments on aphid fecundity, aphid developmental time, and leaf number were assessed by Tukey *post hoc* tests.

The total numbers of *B*. *brassicae* that selected a control leaf vs. a narrow-bandwidth-treated leaf were analyzed by GLM with quasibinomial distribution. Pair-wise comparisons with control plants were carried out for each of the four narrow-bandwidth light treatments.

## Results

### Leaf number, plant height, and weight (without aphid infestation)

**Fig 2:**
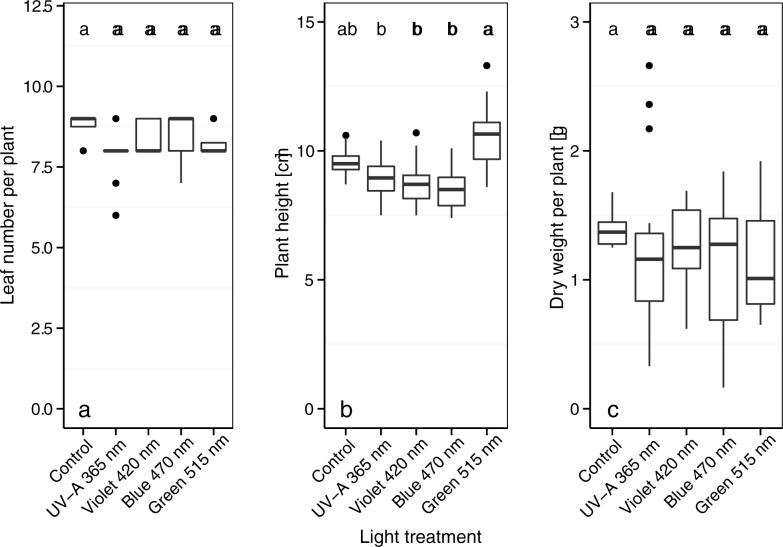
Leaf number (a), plant height, (b) and plant dry weight (c) of 4-week-old broccoli plants grown in a climate chamber and exposed to control lighting plus one of four light treatments. Different letters indicate significant differences (GLM (Fig 2A), GLMM (Fig 2B and 2C), and Tukey *post hoc* tests at *p* < 0.05; *n* = 16 biological replicates).

Leaf number per broccoli plant without aphid infestation was not significantly affected by the light treatments (Fig 2A). Plant height was higher (*p* < 0.01) for plants treated with green light (515 nm) than for plants treated with UV-A light (365 nm), violet light (420 nm), or blue light (470 nm) (Fig 2B). The dry weight of broccoli plants was not affected by the light treatments (Fig 2C).

### Performance and behavior of *B*. *brassicae*

**Fig 3:**
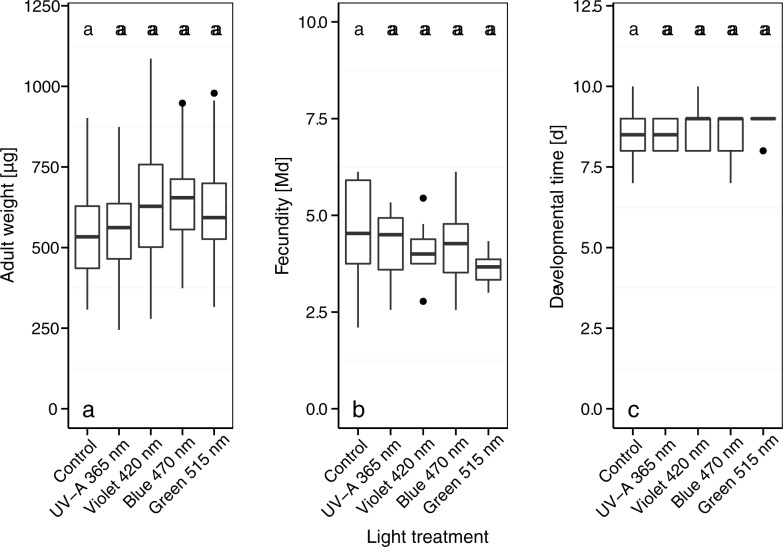
Adult weight (a), fecundity, (b) and developmental time (c) of *B*. *brassicae* kept on broccoli plants that were grown in a climate chamber and exposed to control lighting plus one of four light treatments. Different letters indicate significant differences (GLMM (Fig 3A), GLM (Fig 3B and 3C), and Tukey *post hoc* tests, *p* < 0.05; *n* = 8 biological replicates).

**Fig 4:**
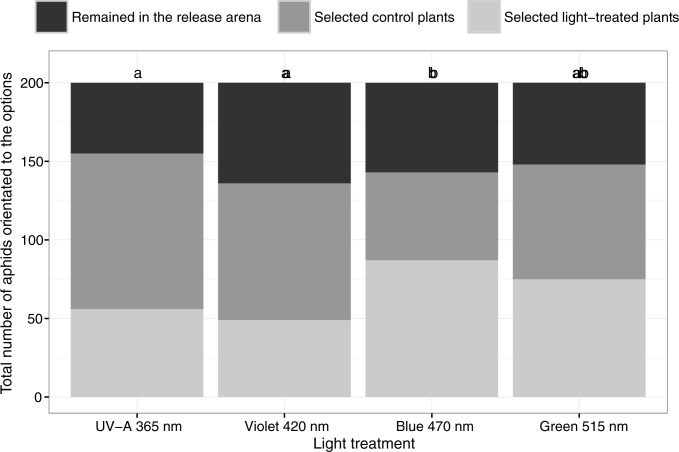
Total number of *B*. *brassicae* adults preferring plants grown under different additional narrow-bandwidth LED treatments or control plants that received only PAR light. Different letters indicate significant differences between light treatments (GLM with quasibinomial distribution and pair-wise comparison of different light treatments, *p* < 0.01; *n* = 20 biological replicates).

Adult weight, fecundity, and developmental time of *B*. *brassicae* were not significantly affected by the light treatments (Fig 3A–3C). The selection of host plant by *B*. *brassicae* was influenced by the light treatments. Significantly more *B*. *brassicae* selected to blue 470 nm-treated plants than UV-A 365 nm-treated plants (*p* < 0.01) or violet 420 nm-treated plants (*p* < 0.01) (Fig 4). The green 515 nm-treated plants tended to be more attractive than control plants but the difference was not significant (*p* < 0.11).

### Hydroxycinnamic acids

**Fig 5:**
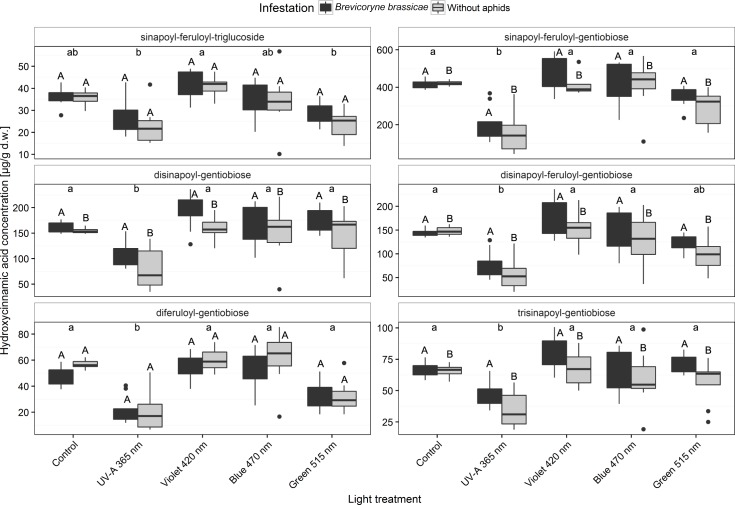
Concentrations of the hydroxycinnamic acids sinapoyl-feruloyl-triglucoside, sinapoyl-feruloyl-gentiobiose, disinapoyl-gentiobiose, disinapoyl-feruloyl-gentiobiose, diferuloyl-gentiobiose, and trisinapoyl-gentiobiose in broccoli plants (infested or non-infested with *Brevicoryne brassicae*) grown in a climate chamber and exposed to control lighting with one of four light treatments or to control lighting without additional illumination. Uppercase letters indicate significant effects of aphid infestation within each light treatment averaged over the level of variant. Lowercase letters indicate significant differences among light treatments averaged across infestation level (GLMM and Tukey *post hoc* tests, *p* < 0.001, *n* = 8 biological replicates).

The following 12 hydroxycinnamic acid derivatives were detected in broccoli plants: caffeoylquinic acid (3-clorogenic acid), caffeoyl-glucoside, sinapoyl-gentiobiose, feruloyl-glucoside, sinapoyl-glucoside, sinapoyl-feruloyl-triglucoside, sinapoyl-feruloyl-gentiobiose (isomer), disinapoyl-gentiobiose, sinapoyl-feruloyl-gentiobiose, diferuloyl-gentiobiose, trisinapoyl-gentiobiose, and disinapoyl-feruloyl-gentiobiose. Among these, the six considered most relevant to the study are listed in Fig 5 and S1 Table. Plants infested with *B*. *brassicae* generally contained increased levels of the monosinaopyl sinapoyl-feruloyl-gentiobiose and of the polysinapoyls disinapoyl-gentiobiose, disinapoyl-feruloyl-gentiobiose, and trisinapoyl-gentiobiose.

The concentration of hydroxycinnamic acids was lowest when broccoli plants were exposed to UV-A light (365 nm) independent of *B*. *brassicae* infestation. Furthermore, quantities of the monosinapoyl sinapoyl-feruloyl-gentiobiose and of the polysinapoyls disinapoyl-gentiobiose, disinapoyl-feruloyl-gentiobiose, and trisinapoyl-gentiobiose did not differ among the control, violet 420 nm, blue 470, and green 515 nm treatments regardless of *B*. *brassicae* infestation (Fig 5 and S1 Table).

### Quercetin glycosides

**Fig 6:**
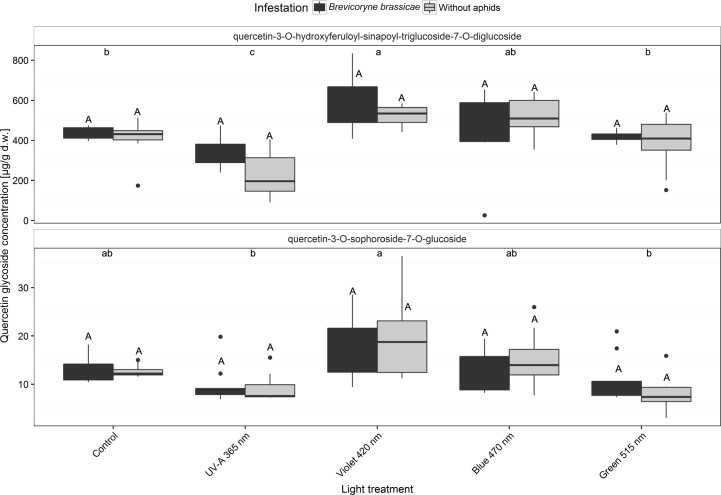
Concentrations of the quercetin glycosides quercetin-3-*O*-hydroxyferuloyl-sinapoyl-triglucoside-7-*O*-diglucoside and quercetin-3-*O*-sophoroside-7-*O*-glucoside in broccoli plants (infested or non-infested with *Brevicoryne brassicae*) grown in a climate chamber and exposed to control lighting with one of four light treatments or to control lighting without additional illumination. Uppercase letters indicate significant effects of aphid infestations averaged over the level of variant. Lowercase letters indicate significant differences among light treatments averaged over the level of infestation (GLMM and Tukey *post hoc* tests, *p* < 0.001, *n* = 8 biological replicates).

The less complex non-acylated quercetin-3-*O*-sophoroside-7-*O*-glucoside and the complex diacylated quercetin-3-*O*-hydroxyferuloyl-sinapoyl-triglucoside-7-*O*-diglucoside were detected in the broccoli plants (Fig 6 and S2 Table). *B*. *brassicae* infestation did not affect the concentrations of quercetin glycosides. The concentrations of quercetin glycosides were lowest in broccoli plants treated with UV-A 365 nm and green 515 nm. Concentrations of quercetin-3-*O*-hydroxyferuloyl-sinapoyl-triglucoside-7-*O*-diglucoside were significantly increased in plants treated with violet 420 nm regardless of *B*. *brassicae* infestation (Fig 6 and S2 Table).

### Kaempferol glycosides

**Fig 7:**
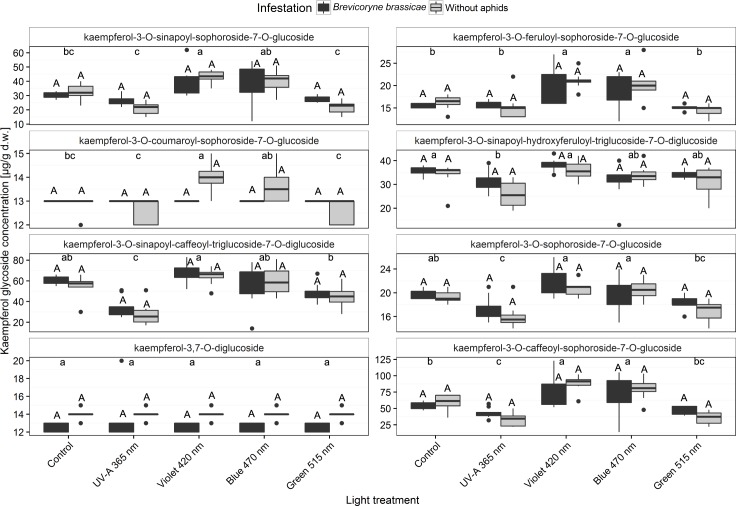
Concentrations of the kaempferol glycosides kaempferol-3-*O*-sinapoyl-sophoroside-7-*O*-glucoside, kaempferol-3-*O*-feruloyl-sophoroside-7-*O*-glucoside, kaempferol-3-*O*-coumaroyl-sophoroside-7-*O*-glucoside, kaempferol-3-*O*-sinapoyl-hydroxyferuloyl-triglucoside-7-*O*-diglucoside, kaempferol-3-*O*-sinapoyl-caffeoyl-triglucoside-7-*O*-diglucoside, kaempferol-3-*O*-sophoroside-7-*O*-glucoside, kaempferol-3,7-*O*-diglucoside, and kaempferol-3-*O*-caffeoyl-sophoroside-7-*O*-glucoside in broccoli plants (infested or non-infested with *Brevicoryne brassicae*) grown in a climate chamber and exposed to control lighting with one of four light treatments or to control lighting without additional illumination. Uppercase letters indicate significant effects of aphid infestations averaged over the level of variant. Lowercase letters indicate significant differences among light treatments averaged over infestation level (GLMM and Tukey *post hoc* tests, *p* < 0.001, *n* = 8 biological replicates).

The following 14 kaempferol glycosides were detected in broccoli plants: kaempferol-3-*O*-hydroxyferuloyl-sophoroside-7-*O*-glucoside, kaempferol-3-*O*-caffeoyl-sophoroside-7-*O*-glucoside, kaempferol-3-*O*-sinapoyl-sophoroside-7-*O*-glucoside, kaempferol-3-*O*-sinapoyl-sophoroside-7-*O*-diglucoside, kaempferol-3-*O*-feruloyl-sophoroside-7-*O*-glucoside, kaempferol-3-*O*-coumaroyl-sophoroside-7-*O*-glucoside, kaempferol-3-*O*-caffeoyl-sophoroside-7-*O*-glucoside (isomer), kaempferol-3-*O*-sinapoyl-hydroxyferuloyl-triglucoside-7-*O*-diglucoside, kaempferol-3-*O*-sinapoyl-caffeoyl-triglucoside-7-*O*-diglucoside, kaempferol-3-*O*-sinapoyl-feruloyl-triglucoside-7-*O*-diglucoside, kaempferol-3-*O*-sinapoyl-feruloyl-triglucoside-7-*O*-diglucoside (isomer), kaempferol-3-*O*-sophoroside-7-*O*-glucoside, kaempferol-3,7-*O*-diglucoside, and kaempferol-3-*O*-glucoside-7-*O*-diglucoside. Among these, eight structurally different compounds were considered most relevant to the study (Fig 7 and S3 Table). *B*. *brassicae* infestation had no effect on the concentration of kaempferol glycosides, which are the main flavonoid glycosides in broccoli. The concentrations of kaempferol glycosides were lowest in UV-A 365 nm- and green 515 nm-treated plants regardless of *B*. *brassicae* infestation. The concentrations of the monoacylated triglycosides kaempferol-3-*O*-sinapoyl-sophoroside-7-*O*-glucoside, kaempferol-3-*O*-feruloyl-sophoroside-7-*O*-glucoside, kaempferol-3-*O*-caffeoyl-sophoroside-7-*O*-glucoside, and kaempferol-3-*O*-coumaroyl-sophoroside-7-*O*-glucoside were significantly increased in broccoli plants treated with violet 420 nm light. Treatment with blue 470 nm light significantly increased concentrations of kaempferol-3-*O*-coumaroyl-sophoroside-7-*O*-glucoside compared with plants grown under control, UV-A 365 nm, or green 515 nm light conditions regardless of *B*. *brassicae* infestation. Concentrations of kaempferol-3-*O*-feruloyl-sophoroside-7-*O*-glucoside and kaempferol-3-*O*-caffeoyl-sophoroside-7-*O*-glucoside were higher in broccoli plants treated with additional blue 470 nm light (Fig 7 and S3 Table).

### Aliphatic glucosinolates

**Fig 8:**
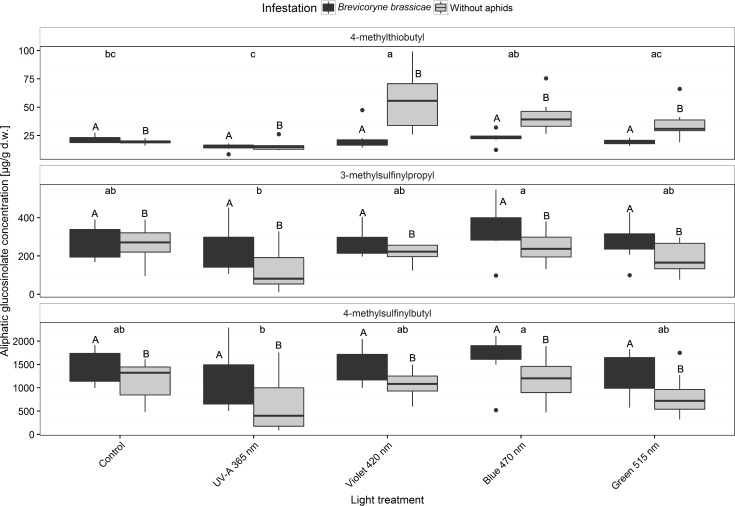
Concentrations of the aliphatic glucosinolates 4-methylthiobutyl, 3-methylsulfinylpropyl, and 4-methylsulfinylbutyl in broccoli plants (infested or non-infested with *Brevicoryne brassicae*) grown in a climate chamber and exposed to control lighting with one of four light treatments or to control lighting without additional illumination. Uppercase letters indicate significant effects of aphid infestations averaged over the level of variant. Lowercase letters indicate significant differences among light treatments averaged over infestation level (GLMM and Tukey *post hoc* tests, *p* < 0.001, *n* = 8 biological replicates).

Three aliphatic glucosinolates (4-methylthiobutyl, 3-methylsulfinylpropyl, and 4-methylsulfinylbutyl) were quantified in the broccoli leaves in all treatments (Fig 8 and S4 Table). The predominant aliphatic glucosinolate was 4-methylsulfinylbutyl. Concentrations of all aliphatic glucosinolates were higher in *B*. *brassicae*-infested plants than in non-infested plants. Regardless of *B*. *brassicae* infestation, the concentration of 4-methylthiobutyl glucosinolate was increased by violet 420 nm light. Concentrations of the methylsulfinylalkyl glucosinolates 3-methylsulfinylpropyl and 4-methylsulfinylbutyl were also increased by blue light both without and with *B*. *brassicae* infestation, but the values were not significantly different from those of the control (Fig 8 and S4 Table).

### Indole glucosinolates

**Fig 9:**
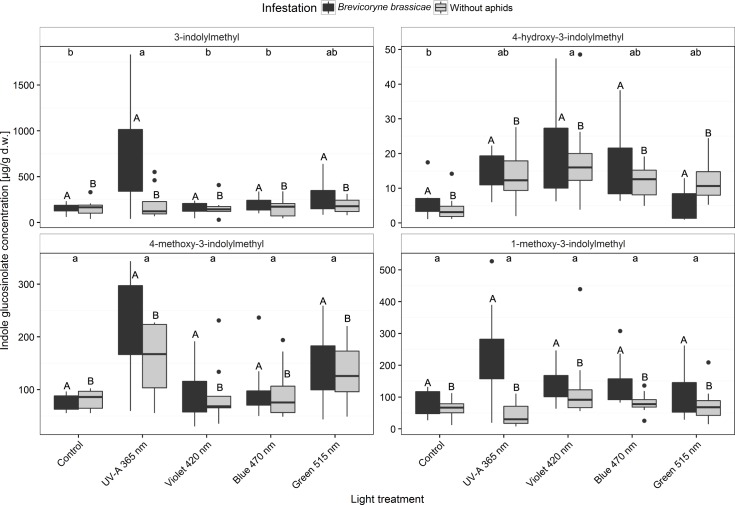
Concentrations of the indole glucosinolates 3-indolylmethyl, 4-hydroxy-3-indolylmethyl, 4-methoxy-3-indolylmethyl, and 1-methoxy-3-indolylmethyl in broccoli plants (infested or non-infested with *Brevicoryne brassicae*) grown in a climate chamber and exposed to control lighting with one of four light treatments or to control lighting without additional illumination. Uppercase letters indicate significant effects of aphid infestation averaged over the level of variant. Lowercase letters indicate significant differences among light treatments averaged over infestation level (GLMM and Tukey *post hoc* tests, *p* < 0.001, *n* = 8 biological replicates).

Four indole glucosinolates (3-indolylmethyl, 4-hydroxy-3-indolylmethyl, 4-methoxy-3-indolylmethyl, and 1-methoxy-3-indolylmethyl) were quantified in the broccoli leaves in all treatments (Fig 9 and S5 Table). Regardless of light treatment, *B*. *brassicae* infestation increased concentrations of all indole glucosinolates. Concentrations of the 3-indolylmethyl glucosinolate were significantly increased by the UV-A 365 nm treatment, particularly with *B*. *brassica* infestation. The concentration of its methoxylated forms, 4-methoxy-3-indolylmethyl glucosinolate and 1-methoxy-3-indolylmethyl glucosinolate, tended to be increased by UV-A 365 nm treatment. The concentration of 4-hydroxy-3-indolylmethyl glucosinolate was significantly increased by violet 420 nm treatment (Fig 9 and S5 Table).

## Discussion

The present study investigated the effects of different narrow-bandwidths of light on the growth of broccoli plants, on the concentrations of glucosinolates and flavonol glycosides in the plants, and on the interaction between the plants and the aphid *B*. *brassicae*, which is a specialized herbivore of *Brassica* spp. We were particularly interested in comparing the effects of short-wavelength light (UV-A) with longer PAR wavelength light (violet, blue, and green).

Broccoli plants grown under additional green light (50 μmol m^-^^2^ s^-1^) in our study were significantly taller than plants grown under the control light or under UV-A, violet, or blue light treatments (Fig 2). Plant responses to green light are typically low-light responses that may help plants grow when under foliage or when near other plants. From a plant perspective, it makes sense to grow taller so as to avoid shade in areas with higher green light intensities [34]. Johkan et al. [18] reported that lettuce plant growth was increased under additional high-intensity green LED light (300 μmol m^-^^2^ s^-1^) with a peak wavelength of 510 nm. In the current study, broccoli plant leaf number and dry weight were unaffected by the light treatments (Fig 2). Fan et al. [35], in contrast, found that Chinese cabbage plants weighed more and were shorter when treated with blue 460 nm LED light than with green 520 nm LED light with intensities of 150 μmol m^-^^2^ s^-1^. These differences between studies demonstrate that the effect of light of different wavelengths can be species-specific.

The LED light treatments had no indirect effect via secondary metabolite composition of the broccoli plant on the performance (adult weight, fecundity, and developmental time) of *B*. *brassicae* (Fig 3). However, shorter wavelengths with a higher amount of energy such as UV-B treatments have been previously shown to increase the concentrations of kaempferol glycosides and indole glucosinolates (3-indolylmethyl and 4-methoxy-3-indolylmethyl) and to reduce the fecundity of *B*. *brassicae* on broccoli plants [6, 22, 25]. In another study, UV-A treatments reduced the reproduction of soybean aphids [36]. Illumination of Brussels sprout plants with additional LED-generated UV-A radiation (259 kJ m^-2^ d^-1^) in a greenhouse increased the concentrations of 3-indolylmethyl glucosinolate in the plants and reduced the fecundity of *B*. *brassicae* relative to blue light-treated plants [37]. In the latter study, 3-indolylmethyl glucosinolate concentrations were as high as 2304 μg g^-1^ d.w. in UV-A treated Brussels sprout plants. The concentrations in the latter study were clearly higher than those in the broccoli plants (207 μg g^-1^ d.w.) that were treated with additional UV-A radiation in a climate-chamber in the present study (Fig 9). This difference might be explained by a plant species-specific difference in sensitivity to UV-A treatments or to a dose-response reaction.

Although some defense compounds reacted, i.e., quercetin-3-*O*-hydroxyferuloyl-sinapoyl-triglucoside-7-*O*-diglucoside and mono-acylated triglycosides of kaempferol were increased by violet light, the concentrations in the present study were quite low and did not significantly affect aphid performance (Figs 6 and 7). Broccoli plants in a previous study that were grown under UV-B treatments contained up to 4100 μg g^-1^ d.w. of single kaempferol glycosides such as kaempferol-3-*O*-caffeoyl-sophoroside-7-*O*-glucoside [6], while broccoli plants treated with longer wavelength in the present study contained low concentrations (< 100 μg g^-1^ d.w.) of all specific kaempferol glycosides even though the illumination times, overall light intensities, and plant stages were the same in both studies (Fig 7). In experiments with Chinese cabbage, Kim et al. [11] also detected very low concentrations of quercetin and kaempferol glycosides (< 10 μg g^-1^ d.w.) after 12 days of illumination with blue, red, or white LEDs.

Plant choice by aphids was indirectly affected by the light treatments in the present study, i.e., significantly more aphids selected plants that had been grown with additional blue light rather than with control light (Fig 4). There was also a non-statistically significant tendency for aphids to prefer plants that had been grown with additional green light rather than with control light, but the aphids showed no preference for plants that had been grown with additional UV-A or violet light (Fig 4). This behavior could only be partly explained by increasing amounts of secondary plant metabolites, because the only enhanced compound was 3-indolylmethyl glucosinolate, which had significantly higher concentrations in plants exposed to UV-A than in plants exposed to blue and violet light treatments. The green peach aphid *Myzus persicae*, on the other hand, preferred Chinese cabbage plants with reduced concentrations of glucosinolates, indicating that secondary plant metabolites could affect host selection by aphids [38]. The indirect effects of light quality on host selection by aphids warrants additional study.

In the choice experiment in the current study, the aphids were able to switch between the two plants after unsuccessful probing on one plant or after determining that one plant was a better nutrient source than the other. Probing by the aphid *Sitobion avenae* was reduced on plants that were treated with enhanced UV-B irradiation as indicated by smaller number of phloem phase, shorter phloem ingestion, and fewer aphids reaching the sustained phloem ingestion phase [39]. Host selection by aphids could also be influenced by host volatiles [40] or by visual cues [41]. To separate between visual or olfactory cues and probing behavior-induced differences in host selection, it would be helpful to conduct olfactory experiments in which aphids did not directly contact the plant.

Blue light can increase the chlorophyll content per leaf area and the photosynthetic rate, resulting in better primary plant metabolism [17, 19]. This could cause host plants to be more attractive to aphids, at least after the initial probing by the aphids. Future studies on host choice by aphids should include the alteration of primary metabolites in the phloem sap.

The light quality of the background spectra can also modify the metabolic composition of a plant, and high PAR intensities with a high amount of blue light can improve photosynthetic performance and acclimatization to and recovery from UV irradiation [19, 42]. The background spectrum contained more blue light but less red light in the current study (Fig 1) than in our previous study [6], although the light intensity was the same (100 µmol m^-^^2^ s^-1^ PAR) in both studies. The induction of secondary metabolites by PAR may provide a basic level of UV protection that is optimized and increased by UV-B and UV-A radiation [43]. Concentrations of secondary plant metabolites (such as glucosinolates and flavonol glycosides) in broccoli plants grown in a climate chamber with specific UV-B, UV-A, and violet light treatments were greater with more red light in the background spectrum [6] than with more blue light in the background spectrum (as in the current study). This could partially explain the differences in aphid performance between these two studies, but the effect of the background spectrum was not investigated in detail in either study. Shorter wavelength (UV-A) light as well as longer PAR wavelength (violet to green) light in combination with a blue background spectrum were unable to sufficiently alter the concentrations of glucosinolates and flavonol glycosides so as to reduce the performance of *B*. *brassicae* on broccoli plants in the present study. Future studies should carefully consider the effect of differences in background light quality and quantity.

## Conclusion

In conclusion, this study has demonstrated that similar intensities of narrow-bandwidth light treatments in addition to PAR can alter the concentration of specific secondary metabolites in broccoli plants. The concentrations of flavonol glycosides and glucosinolates in this study were quite low and did not affect the performance of the specialized aphid *B*. *brassicae*. Host choice by *B*. *brassicae* was indirectly influenced by the narrow-bandwidth light treatments in that the aphid preferred blue light-illuminated plants (but not UV-A-, violet-, or green-illuminated plants) to control plants.

In future studies, insect feeding assays should be used to determine the concentrations of primary or secondary plant metabolites necessary to influence host choice and population increase of target herbivores. Future studies should also investigate whether higher LED intensities, optimal illumination times, and combinations of light qualities can increase the concentrations of secondary plant metabolites so as to protect greenhouse cultured plants against insect herbivores.

## Supporting information

S1 Table Concentrations [µg g^-1^ d.w.; mean (± SE)] of hydroxycinnamic acids in broccoli plants (non-infested, or infested with *B*. *brassicae*) grown in a climate chamber and exposed to control lighting plus one of four light treatments or to control lighting without additional illumination. Uppercase letters indicate significant effects of aphid infestation averaged over the level of variant. Lowercase letters indicate significant differences of light treatments averaged over the level of infestation (GLMM and Tukey *post hoc* tests, *P* < 0.001, *N* = 8 biological replicates). Sin-Fer-Triglc: sinapoyl-feruloyl-triglucoside, Sin-Fer-Gent: sinapoyl-feruloyl-gentiobiose, Disin-Gent: disinapoyl-gentiobiose, Disin-Fer-Gent: disinapoyl-feruloyl-gentiobiose, Difer-Gent: diferuloyl-gentiobiose, Trisin-Gent: trisinapoyl-gentiobiose.
(DOCX)

S2 Table Concentrations [µg g^-1^ d.w.; mean (± SE)] of quercetin glycosides in broccoli plants (non-infested, or infested with *B*. *brassicae*) grown in a climate chamber and exposed to control lighting plus one of four light treatments or to control lighting without additional illumination. Uppercase letters indicate significant effects of aphid infestations averaged over the level of variant. Lowercase letters indicate significant differences of light treatments averaged over the level of infestation (GLMM and Tukey *post hoc* tests, *P* < 0.001, *N* = 8 biological replicates). Q-3-hfer-sin-triglc-7-diglc: quercetin-3-*O*-hydroxyferuloyl-sinapoyl-triglucoside-7-*O*-diglucoside, Q-3-soph-7-glc: quercetin-3-*O*-sophoroside-7-*O*-glucoside.
(DOCX)

S3 Table Concentrations [µg g^-1^ d.w.; mean (± SE)] of kaempferol glycosides in broccoli plants (non-infested, or infested with *B*. *brassicae*) grown in a climate chamber and exposed to control lighting plus one of four light treatments or to control lighting without additional illumination. Uppercase letters indicate significant effects of aphid infestations averaged over the level of variant. Lowercase letters indicate significant differences of light treatments averaged over the level of infestation (GLMM and Tukey *post hoc* tests, *P* < 0.001, *N* = 8 biological replicates). K-3-sin-soph-7-glc: kaempferol-3-*O*-sinapoyl-sophoroside-7-*O*-glucoside, K-3-fer-soph-7-glc: kaempferol-3-*O*-feruloyl-sophoroside-7-*O*-glucoside, K-3-cou-soph-7-glc: kaempferol-3-*O*-coumaroyl-sophoroside-7-*O* glucoside, K-3-sin-hfer-triglc-7-diglc: kaempferol-3-*O*-sinapoyl-hydroxyferuloyl-triglucoside-7-*O*-diglucoside, K-3-sin-caf-triglc-7-diglc: kaempferol-3-*O*-sinapoyl-caffeoyl-triglucoside-7-*O*-diglucoside, K-3-soph-7-glc: kaempferol-3-*O*-sophoroside-7-*O*-glucoside, K-3-7-diglc: kaempferol-3,7-*O*-diglucoside, kaempferol-3-*O*-sophoroside-7-*O*-glucoside, K-3-caf-soph-7-glc: kaempferol-3-*O*-caffeoyl-sophoroside-7-*O*-glucoside.
(DOCX)

S4 Table Concentrations [µg g^-1^ d.w.; mean (± SE)] of aliphatic glucosinolates in broccoli plants (non-infested, or infested with *B*. *brassicae*) grown in a climate chamber and exposed to control lighting plus one of four light treatments or to control lighting without additional illumination. Uppercase letters indicate significant effects of aphid infestations averaged over the level of variant. Lowercase letters indicate significant differences of light treatments averaged over the level of infestation (GLMM and Tukey *post hoc* tests, *P* < 0.001, *N* = 8 biological replicates).
(DOCX)

S5 Table Concentrations [µg g^-1^ d.w.; mean (± SE)] of indole glucosinolates in broccoli plants (non-infested, or infested with *B*. *brassicae*) grown in a climate chamber and exposed to control lighting plus one of four light treatments or to control lighting without additional illumination. Uppercase letters indicate significant effects of aphid infestations averaged over the level of variant. Lowercase letters indicate significant differences of light treatments averaged over the level of infestation (GLMM and Tukey *post hoc* tests, *P* < 0.001, *N* = 8 biological replicates).
(DOCX)
