# Supplementary material for: Can narrow-bandwidth light from UV-A to green alter secondary plant metabolism and increase Brassica plant defenses against aphids?
Source: PLoS One. 2017 Nov 30;12(11):e0188522. doi: 10.1371/journal.pone.0188522 (PMC5708618; doi:10.1371/journal.pone.0188522)
Supplement: S3 Table — Uppercase letters indicate significant effects of aphid infestations averaged over the level of variant. Lowercase letters indicate significant differences of light treatments averaged over the level of infestation (GLMM and Tukey post hoc tests, P < 0.001, N = 8 biological replicates). K-3-sin-soph-7-glc: kaempferol-3-O-sinapoyl-sophoroside-7-O-glucoside, K-3-fer-soph-7-glc: kaempferol-3-O-feruloyl-sophoroside-7-O-glucoside, K-3-cou-soph-7-glc: kaempferol-3-O-coumaroyl-sophoroside-7-O glucoside, K-3-sin-hfer-triglc-7-diglc: kaempferol-3-O-sinapoyl-hydroxyferuloyl-triglucoside-7-O-diglucoside, K-3-sin-caf-triglc-7-diglc: kaempferol-3-O-sinapoyl-caffeoyl-triglucoside-7-O-diglucoside, K-3-soph-7-glc: kaempferol-3-O-sophoroside-7-O-glucoside, K-3-7-diglc: kaempferol-3,7-O-diglucoside, kaempferol-3-O-sophoroside-7-O-glucoside, K-3-caf-soph-7-glc: kaempferol-3-O-caffeoyl-sophoroside-7-O-glucoside. (DOCX) [file pone.0188522.s003.docx]

| **Kaempferol glycosides** | **Light treatment and concentrations of kaempferol glycosides** | | | | |
| --- | --- | --- | --- | --- | --- |
| **Without aphid infestation** | **Control** | **UV-A 365 nm** | **Violet 420 nm** | **Blue 470 nm** | **Green 515 nm** |
| K-3-sin-soph-7-glc^(A)^ | 33 ± 5^(bc)^ | 21 ± 4^(c)^ | 43 ± 4^(a)^ | 40 ± 8^(ab)^ | 22 ± 4^(c)^ |
| K-3-fer-soph-7-glc^(A)^ | 16 ± 2^(b)^ | 15 ± 3^(b)^ | 21 ± 2^(a)^ | 20 ± 4^(a)^ | 14 ± 1^(b)^ |
| K-3-cou-soph-7-glc^(A)^ | 13 ± 1^(bc)^ | 13 ± 1^(c)^ | 14 ± 1^(a)^ | 14 ± 1^(ab)^ | 13 ± 1^(c)^ |
| K-3-sin-hfer-triglc-7-diglc^(A)^ | 34 ± 5^(a)^ | 26 ± 6^(b)^ | 36 ± 4^(a)^ | 34 ± 4^(ab)^ | 31 ± 7^(ab)^ |
| K-3-sin-caf-triglc-7-diglc^(A)^ | 55 ± 11^(ab)^ | 28 ± 11^(c)^ | 64 ± 8^(a)^ | 60 ± 13^(ab)^ | 45 ± 10^(b)^ |
| K-3-soph-7-glc^(A)^ | 19 ± 1^(ab)^ | 16 ± 2^(c)^ | 21 ± 1^(a)^ | 21 ± 2^(a)^ | 17 ± 2^(bc)^ |
| K-3-7-diglc^(A)^ | 14 ± 1^(a)^ | 15 ± 1^(a)^ | 14 ± 1^(a)^ | 15 ± 1^(a)^ | 16 ± 1^(a)^ |
| K-3-caf-soph-7-glc^(A)^ | 33 ± 5^(b)^ | 21 ± 4^(c)^ | 43 ± 4^(a)^ | 40 ± 8^(a)^ | 21 ± 4^(bc)^ |
| **With *B. brassicae*  infestation** |  |  |  |  |  |
| K-3-sin-soph-7-glc^(A)^ | 30 ± 2^(bc)^ | 26 ± 4^(c)^ | 41 ± 10^(a)^ | 38 ± 14^(ab)^ | 28 ± 2^(c)^ |
| K-3-fer-soph-7-glc^(A)^ | 16 ± 1^(b)^ | 16 ± 1^(b)^ | 2 ± 4^(a)^ | 19 ± 4^(a)^ | 15 ± 1^(b)^ |
| K-3-cou-soph-7-glc^(A)^ | 13 ± 1^(bc)^ | 13 ± 1^(c)^ | 14 ± 1^(a)^ | 14 ± 1^(ab)^ | 13 ± 1^(c)^ |
| K-3-sin-hfer-triglc-7-diglc^(A)^ | 36 ± 2^(a)^ | 31 ± 5^(b)^ | 38 ± 3^(a)^ | 31 ± 8^(ab)^ | 34 ± 2^(ab)^ |
| K-3-sin-caf-triglc-7-diglc^(A)^ | 61 ± 4^(ab)^ | 34 ± 11^(c)^ | 68 ± 10^(a)^ | 57 ± 21^(ab)^ | 48 ± 9^(b)^ |
| K-3-soph-7-glc^(A)^ | 20 ± 1^(ab)^ | 17 ± 2^(c)^ | 22 ± 3^(a)^ | 20 ± 3^(a)^ | 18 ± 1^(bc)^ |
| K-3-7-diglc^(A)^ | 13 ± 1^(a)^ | 13 ± 1^(a)^ | 13 ± 1^(a)^ | 14 ± 1^(a)^ | 14 ± 1^(a)^ |
| K-3-caf-soph-7-glc^(A)^ | 30 ± 2^(b)^ | 26 ± 3^(c)^ | 41 ± 10^(a)^ | 37 ± 13^(a)^ | 27 ± 2^(bc)^ |
